# To: Posterior reversible encephalopathy syndrome in a child with severe multisystem inflammatory syndrome due to COVID-19

**DOI:** 10.5935/2965-2774.20230322-en

**Published:** 2023

**Authors:** Sounira Mehri, Josef Finsterer, Sinda Zarrouk

**Affiliations:** 1 Biochemistry Laboratory, Nutrition-Functional Foods and Vascular Health, Faculty of Medicine, University of Monastir - Monastir, Tunisia; 2 Neurology and Neurophysiology Center - Vienna, Austria; 3 Pasteur Institute of Tunis - Tunis, Tunisia

## To the editor

We eagerly read the article by Dominguez-Rojas et al. about a 9-year-old male with a 3-day history of a gastrointestinal infection who underwent explorative abdominal surgery for acute abdomen, which was noninformative.^(1)^ Postoperatively, the patient developed pneumonia requiring mechanical ventilation.^(1)^ After extubation, the patient was diagnosed with multisystem inflammatory syndrome in children (MIS-C) based on the presence of elevated immunoglobulin G (IgG) antibodies against severe acute respiratory syndrome coronavirus 2 (SARS-CoV-2) and with posterior reversible encephalopathy syndrome (PRES), which was regarded as causally related to SARS-CoV-2.^(1)^ The study is attractive but carries limitations that raise concerns that should be extensively discussed.

The main limitation of the study is that a causal association between PRES and SARS-CoV-2 was established, but the diagnosis remained unconfirmed. The index patient tested negative for SARS-CoV-2 by polymerase chain reaction (PCR). Only IgG antibodies were elevated. The patient was not tested for SARS-CoV-2 by PCR on bronchial secretions during mechanical ventilation.^(1)^ There was no determination of the cytokine or chemokine levels in the serum. Since anti-SARS-CoV-2 IgG can persist for months,^(2)^ a causal relation between PRES and COVID-19 is highly speculative.

A further limitation is that the patient underwent gastrointestinal surgery because of an obvious gastrointestinal infection.^(1)^ Was there evidence of paralytic ileus or mechanical obstruction? Explorative surgery is not usually the treatment of choice for acute abdomen. What conservative measures were taken to heal the acute abdominal complaints?

Another limitation is that cerebrospinal fluid studies were not performed, so several differential diagnoses were not excluded. These include infectious diseases, immune encephalitis, and acute disseminated encephalomyelitis. Cerebrospinal fluid was not available for the determination of cytokines, chemokines, and SARS-CoV-2 RNA. Has venous sinus thrombosis been ruled out by magnetic resonance venography? The D-dimer level was significantly elevated, suggesting thrombosis.

The patient was diagnosed with “signs of coagulopathy”.^(1)^ We should know if deep venous thrombosis, venous sinus thrombosis, and pulmonary embolism have been diagnosed and if evidence of thrombosis other than elevated D-dimer and prolonged prothrombin time has been identified.

The patient had obviously developed heart failure.^(1)^ We should be informed about the cause of heart failure. Was it due to pneumonia, pulmonary embolism, Takotsubo syndrome, or myocarditis? What were the results of the electrocardiogram and the creatine-kinase levels? Because the patient had elevated troponin levels,^(1)^ it is crucial that myocardial infarction, Takotsubo syndrome, and myocarditis were carefully ruled out.

The sodium reference limit of < 135mEq/L is unusual.^(1)^ According to this limit, the patient had hypernatremia. Are the reference limits correct? How was hypernatremia managed?

Because PRES is commonly associated with elevated blood pressure,^(3)^ readers should know if the patient’s blood pressure was ever elevated during treatment in the pediatric intensive care unit.

Overall, this interesting study has some limitations that challenge the results and conclusions. Clarifying these shortcomings would strengthen the conclusions and make the study more compelling. Before SARS-CoV-2 is blamed as the cause of PRES, several differential diagnoses need to be ruled out.
